# On Disorders of Assimilation, Digestion, Etc.

**Published:** 1903-09

**Authors:** 


**On Disorders of Assimilation, Digestion, etc.** By Sir LAUDER BRUNTON, M.D., D.Sc., F.R.S. Pp. xx., 495. London: Macmillan and Co. Ltd. 1901.

The author explains that this is in a sense a second series of papers and addresses on Disorders of Digestion, and that being a collection the same ideas recur again and again. Nevertheless, the matter, even when not new, is presented in different language, and oftentimes from different standpoints, yet always with that clearness and simplicity that characterises Lauder Brunton's handling of scientific topics, and makes intricate scientific questions so attractive to the reader.

Perhaps the most useful to the practitioner will be the series of articles on Diabetes, and the Influence of Stimulants and Narcotics on Health. But every one of the many other articles is worth careful reading, and cannot fail to instruct.

